# The Miracle of Anæsthesia

**Published:** 1861-06

**Authors:** Frank W. Reilly

**Affiliations:** Chicago


ARTICLE III.

### "THE MIRACLE OF ANÆSTHESIA."

### AN INAUGURAL THESIS.

By FRANK W. REILLY, M. D., Chicago.

The doubting Disciple, whose vision required the corroborative testimony of his touch, has, unquestionably, far outstripped his brethren in the number and zeal of his followers. The words of the Hebrew preacher, in their most literal and isolated sense, embody their creed: *We believe* "There is no new thing under the sun;" and to them any attempt to change the manners and customs of their fathers comes in such questionable guise as to warrant their most active incredulity. Unfortunately, too, no experience is impressive enough to be lasting in their doubtful souls; but convinced, if convinced at all, "against their wills, they are of the same opinion still," with reference to every new phase presented by human progress. Faith, even as a grain of mustard seed, is omitted in their constitutions; and we are finally obliged, so widely-spread and so ineradicable is the evil, to admit that antagonism to the New, and blind adherence to the Old, is as much the normal condition of some minds, as is, of others, a healthy progress towards the true and right, regardless of the ruts and grooves of time-honored custom. And the proposition is equally true of all the various branches of human industry, science or art—alike in Medicine and Mechanics.

I have termed this opposition an *evil*,—and yet it may have its important uses—as leading to fuller elucidation, more thorough research—killing off pretension and sham—a sort of natural psychological agency, cutting off those children of the brain, born below that minimum of value, which is a condition of scientific viability, as the Anatomist of Harvard might say. This, however, I do not propose to discuss here, content to know that the history of Medicine furnishes instances of more determined and unscrupulous hostility to innovation, than that of any other Science or Art. The early history of the Circulation of the Blood, with its chain of contradictions and corrections, linking the centuries, furnishes a fair illustration:—Erasistratus, Galen, Vesalius, Servetus, Columbus, Cesalpinus, Fabricius and his immortal pupil, Harvey—each a step in advance of his predecessor, and all a subject of ridicule, abuse and persecution for that step. The following quotations from the cotemporaneous literature of Harvey, may be interesting and novel enough to warrant their introduction and excuse their length:

Guy Patin, Dean of the Faculty of Paris, about 1660, and who has been recently complimented, possibly justly, as "a wit, and a man of sense and learning," wrote as follows: "If M. Duroyer knows nothing more than how to lie, and the circulation of the blood, his knowledge is limited to two things, of which I hate one, and do not care for the other. Let him come to me and I will teach him a better way to a good practice of medicine, than this pretended circulation." "A wit," certainly,—but "a man of sense"!? And his colleague, Riolan, whom Bartholin called the *greatest* anatomist of the age, wrote to a friend, as follows, *apropos* of the then newly announced discovery of the lacteal circulation:—"Every one must now be making discoveries. Pecquet has done worse; for by his new and unheard-of doctrine of the lacteals and the thoracic duct, he will upset both the ancient and modern systems of medicine,"—and more to the same effect. Indeed, we find this estimable Faculty of Paris, this Medical Inquisition, arraigning before it every unfortunate wight who dared be so much the master of his own brain and its research as to question their infallibility, or to protest against the dogmas of their immaculate conception. Opium, antimony, cinchona, even tea, fell under their ban—this last being characterized as an impertinent innovation. Those who dared exhibit antimony were tried and condemned by the Faculty, a proceeding, which in the words of the learned Dean,—"brought them back to their senses. If," continues Doctor Patin, "they should be again wanting in their duty, *we* shall not be in *ours;* but shall proceed summarily against them, so that they will be forever expelled from amongst us."

I may not stop to dwell upon the abuse heaped upon those who undertook to check the use of the lancet, by the disciples of Dr. Sangrado—of whom this very Patin was one of the most ultra, bleeding one patient *thirty-two* times in one attack; himself *seven times* for a cold; his wife *eight times in the arm and then in the foot*, and his mother-in-law only *four times*, *because* she was eighty years old! "We save more patients with a good lancet and senna, than were ever saved by the Arabian physicians with all their syrups and opiates." What a fearfully fatal practice the Arabian must have been!

Again, the warfare against small-pox inoculation, by which the mortality of that dreadful scourge, was reduced from *one in four*, to one *in three or four hundred*, enlisted some of the most eminent members of the clerical and medical professions —the one urging its impiety, and the other its impropriety and danger; and so violent was the feeling that Lady Montagu protests that she "seldom passed a day in the first four or five years of the practice, without repenting her patriotic undertaking," and that she would "never have attempted it, if she had foreseen the vexation, persecution, and even the obloquy it brought upon her."

Half a century later, Jenner, the immortal, whose lancet, it is claimed, saved more lives than the sword of the first Napoleon destroyed, aroused such a storm of indignation, ridicule and abuse, that clergy, physicians and laity were alike carried away by it—the King, Parliament and Faculties implored to interfere against the "destructive practice of vaccination"—"a gross violation of religion, morality, law and humanity"—and personal violence, even, was offered to its advocates. In Materia Medica, the introduction of nearly every important agent; in Surgery the ligation of arteries, the flap operation, resection of the hip-joint, etc.; in another branch, the induction of premature labor and craniotomy, as conservative operations. In short, the history of the advent of all great new movements in medicine, as in other sciences, includes, if complete, the record of opposition and argument, so violent and illogical, so obstinate and absurd, as to excite the pity, wonder and ridicule of succeeding generations—themselves, but too often, re-enacting similar scenes.

Arguments, illogical and absurd, have been used in all ages by the opponents of Progress; but it has been reserved to this Nineteenth Century to urge the *desireability and beneficence of pain*, as an argument against the introduction of agents to annul it,—nay, to such extremes have the opponents of this "glad and glorious discovery," this "Miracle of Anæsthesia" been driven, that, subsequently, they have tacitly, if not expressly denied the existence of Pain! reminding one in the sequence and congruity of their premises, of the defence of the woman, accused of returning her neighbor's tub in a damaged condition: Firstly, the bottom was out when she borrowed the tub; and secondly, she never had the tub at all!

In the early day of this opposition, and when Anæsthesia was restricted to Surgery, such eminent names as Magendie, Syme, Cooper, Condie, and many others of less note were found advancing such arguments. The distinguished French physiologist thought it "a trivial matter to suffer," and that "pain has always its usefulness." Syme thought the pain occasioned, even by the most severe operations, the removal of tumors, amputations, lithotomy, etc., was "nowise unbearable"; indeed he gravely remarks in his report of a case of lithotomy, that "the fatal result was the effect of the removal of a source of extreme irritation in a very irritable system,"—in other words, the pain kept the man alive, and he died when it was removed! The medical literature immediately succeeding the announcement of Dr. Morton's discovery, or application of a previous discovery, teems with articles reflecting such sentiments; but thanks to such earnest workers and friends of humanity as Simpson and Miller of Edinburgh, Liston, Bell and Snow of London, Flourens, Velpeau and Roux of Paris, Mutter, Pancoast, Gross, Mott, Carnochan, Mussey and others in the United States, such a position has long been abandoned; and while the domain of operative interference has been almost indefinitely extended, its mortality has been decreased, its dread paraphernalia of glittering steel and fearful engine, robbed of former terrors, and Surgery is now no long a synonyme for Suffering.

If the record, ending here, were complete, humanity might congratulate itself, and the medical profession point proudly to this, as one of the brightest pages in its annals. Unfortunately, causes, in themselves, let us hope, temporary and inadequate, have combined to limit, almost exclusively, the use of Anæsthetic agents to the operating table; and in these pages I have proposed to myself to briefly examine these causes, to consider the arguments advanced against Anæsthesia in Obstetrics, and to again present the rebutting testimony of those large minded, active sympathizers with all human suffering, who fully realize the Baconian definition of the true physician—whose "office it is, not only to restore health, but to mitigate pains and dolours."

I trust I appreciate in some degree the magnitude of the task, and the inadequacy of the instrument; but I reflect that forces are not necessarily commensurate with results, and am further assured by the belief that a reaction is taking place in the obstetrical mind on this point. In proof of this last, I may cite the fact that Prof. Ramsbotham, of London, whose name is classic in his branch, so far modifies his opinion in 1856, as to omit, in a great measure, the unequivocal proscription which characterized his dictum of 1851; and it is but fair to presume, in view of this known change, that he has since, still further become convinced of the untenable character of his position. Of the work of Prof. Miller, of Louisville, the result of the experience of over a third of a century's ministration "at the altar of Lucina," and nearly ten years' administration of Chloroform, I need not speak, save to allude to the marked and increasing favor with which it is being received by the profession. That I do not allude more fully to the immortal discoverer of the process, J. Y. Simpson, of Edinburgh, is due to the fact that his position is fully recognized and undisputed—my purpose here, being to show the changes taking place, either in teachers themselves, or in the reception of their instructions.

The less grateful duty of examining the causes of proscription, involves the highest didactic authority of America—for to this proud pre-eminence in midwifery and its collateral topics

2

has Prof. Chas. D. Meigs, of Philadelphia, been assigned both by his own countrymen and by the profession of Europe. His position as a teacher, not less than his reputation for learning, skill and success, has been used persistently and uncompromisingly to the exclusion of the wondrous vapor from those who travail in child-birth,—with what success, let the agonized groans and shrieks from the lying-in chambers of five millions American mothers bear witness.

Filling the chair of his speciality in the leading college of the country, with annual classes of over five hundred eager, credulous students, and annual graduates numbering over two hundred,—in the twelve years of his hostility he has sent out upwards of twenty-five hundred practitioners and over six thousand students, imbued with the belief that the administration of chloroform to the induction of insensibility to pain in parturient women, is not only fraught with mischief and danger, but is a violation of both morals and religion. When to professorial influence is added the multiplied editions of his text-books, forming, no doubt, the entire obstetrical literature of numerous practitioners,—his further influence as a clinical instructor, and as member or officer of numerous other organizations, in all of which he is brought in contact with new minds, only too willing to receive, from his personal presence, courteous and winning to a degree, a favorable bias towards one of whom so much is said,—when all this is duly considered, is it any wonder that he should have succeeded in moulding the sentiment of the profession, on this point, so thoroughly as he has done?

Assuming this to be true, and that to the Obstetrican of Philadelphia is due the tabooing of this Miracle of the Age, it becomes necessary, first to examine, briefly, the weight and importance of his arguments against the practice; and secondly, to answer them as well as I may, in the light of the observations and experience of its advocates.

Before doing so, however, it may be but simple justice to notice an argument, which may be advanced by those seeking to deny the charge that Prof. Meigs has been allowed to do their thinking for them, or that they have always unquestioningly pinned their faith upon his sleeve, viz: the fact that the profession ignores the learned Dr.'s theory of the "*Heart Clot*," and that his definitions and theories of Conception, Pregnancy, etc., given with so much emphasis of spaced words and epigrammatic diction, are taken only *cum grano salis*. Grant that the profession does not endorse these and kindred views; the very promulgation of such views proves the fallibility of their author; and if Prof. Meigs could issue and adhere to such a theory as that of the "*Heart Clot*," based, as it is, upon a palpable misapprehension of an effect for a cause; or could advance such definitions of two of the most important functions of 'the sex' as to logically yield such paradoxes as these:—*a woman may be pregnant before she conceives; abortion may occur before conception*,—if these are among his teachings, why may he not be equally in error regarding a matter, which he positively refuses to investigate?

The objections of Prof. Meigs to the induction of Anæsthesia in Parturition, as embodied in 1848, in a letter to Prof. Simpson, and as subsequently set forth, in connection with said letter, in his "Science and Art," may be summed up under two heads; 1st, *Its undesireability and impropriety;* and 2d, *Its danger and evil results;* and these include the items of all the arguments of its opponents worth notice. Indeed, I might very properly, and perhaps profitably, omit any notice of the counts under the first head; but from the prominence which has been given them, from time to time, I prefer a brief consideration of them.

The opponents of Anæsthesia allege, firstly, and most prominently, that its induction is undesirable and improper, for that the pain of labor "is a most desirable, salutary and conservative manifestation of life-force," (*Meigs*): and is "an essential element in the function of parturition," (*Ramsbotham*).

The first may be admitted, in part; the second, denied *in toto*.

That "an infinite Wisdom has contrived pain for our protection," has made it "the grand preserver of existence," "the sleepless sentinel that watches over our safety," I very readily admit. The burnt child that dreads the fire, and the juvenile Davy, lustily kicking off the historic crustacean that would dine upon his toes, are alike the subjects of its beneficent, though apparently severe dominion; the "manifestation of life-force" in both instances, is, indeed, "most desirable, salutary and conservative."

The pain of parturition, however, is a "manifestation of life-force," "desirable, conservative and salutary" to this extent, no more: That it informs us, in the first stage, that the contractions of the fundus and body are pressing the child's head upon the neck and mouth; it is simply an index of the fitful conflict going on between the "contentive and expulsive faculty" of the former and the "retentive faculty" of the latter; and no more an "essential element in the function of parturition," than the clatter and noise of the printing press is an "essential element" of the diffusion of knowledge. In the second stage it possesses hardly this doubtful merit; and to urge any claim to favorable consideration of it in the third stage, is to imply that those fortunate women who escape without "after-pains," fail (to use a homely but very Yankee figure), "to get the worth of their money." These pains are not alone useless, but pernicious since they do not fulfil the legitimate office of Pain, which, according to the highest authority on the subject, Sir Charles Bell—before quoted, is that of Warning—warning that tissues are endangered, the integrity of organs threatened—warning to the end that the danger may be averted and relief afforded. But relief, in labor, can be afforded only by Nature in the slow process of the removal of the foreign body—the fœtus. Therefore, the pains are useless; and inasmuch as they tend to excite, exhaust and depress the patient they are pernicious. Why, then, not annul these pains—useless, yet so injurious? Oh, because, say the opponents of Anæsthesia, their wits sharpened by the force of necessity, because Pain is two-fold, *physiological* and *pathological;* and the latter only comes within the province of the physician. And so, labor-pains being the result of the "culmination of the female somatic forces," hence, "physiological," it is improper to interfere with them. And, besides, in this new view of the subject, they don't hurt near so much—at least, Prof. Meigs don't feel them so acutely, as when he wrote, with the sentimental *näiveté* of a Michelêt his chapter on Labor and his Letters to his Class, from whence these extracts: "And the word (*Labor*), is highly expressive of the fatiguing, violent and painful struggles and efforts." "The sensation, under these circumstances (the last expulsive pains) is represented as absolutely indescribable, and, certainly, as comparable to no other pain." And again, when, in a generous glow of tender gallantry, he asserts that "Man cannot suffer the same pains as Woman"—and querying "What do you call the pain of parturition?"—justly answers, "There *is* no name for it, but *Agony!*"

This is certainly clear and unmistakeable; yet let us once again quote from the same high authority, and this time as touching our modern Cartesian doctrine of Pain:

"The pain of a labor consists chiefly, though not solely, in the pain felt at the cervix uteri, and is the result of *violence* done to the *texture* of the cervix and other parts, etc."

"If a man strikes you a blow with his fist, and knocks you down, he hurts you and not himself; in the same way the fundus and the corpus uteri, when they strike to rive open and overcome the resistance of the cervix and os uteri, *hurt* them, *excite pain* in them, and sometimes even *tear them to pieces*," etc.

Very well; but how does this violence, these blows, riving open and overcoming, hurting and exciting, even tearing to pieces, these exquisitely sensitive textures, how does this description comport with the following, written since Anæsthesia has so modified Prof. Meigs' views, and awakened to a spasmodic, ephemeral vitality, the Rowleys, Moseleys and Munroes of Jenner's time, and the scores of others, of every age, saved only from a charitable oblivion, by the unenviable notoriety which attaches to them, as opponents of the progress of their respective periods: "I contend that it is to an exaggerated notion of the nature of labor-pains, we owe the too frequent use of ether in our art." "The representations that have been made by the friends of Anæsthesia, of the harrowing distress endured by women in childbirth, do not consist with the general state of facts in the case." And again: "But in midwifery, to which a long and extensive practice has enured me, and rendered me a familiar, dispassionate witness of its various forms and phenomena * * * * I have found that women provided," etc., etc. I wish to observe here, that Professor Meigs claims only to have found that "women, sustained by cheering counsel and promises, and carefully freed from the distressing element of terror, could, in *general*, be *made to endure* without GREAT *complaint.*" A proposition so carefully guarded and limited by provisions and conditions, that its apparent force is very materially weakened to the careful reader. The first extracts, however, were written before the Anæsthetic innovation was dreamed of, even by the most sanguine; and Prof. Meigs, in his later position, as "a familiar and dispassionate witness," may, in the words of a distinguished Illinois Senator, "object to bringing in issues of individual consistency." But until he reconciles his dicta, or shows ground for the rejection of his former teachings, endorsed by the profession, and harmonizing with the views of all leading authorities, we must admit Labor-Pain to be as severe, and injurious, and as legitimately the object of the physician's concern, as the pain caused by any other, the most purely *pathological.*

Well, but, says Prof. Meigs, the patient's ability to counsel the operator in instrumental cases, is too valuable to be abrogated; the "patient's answer, 'yes' or 'no' to the accoucheur's query 'does it (the forceps) hurt you?' is worth a thousand dogmas and precepts as to planes and axes and curves of Carus." To treat this argument seriously, would be to impeach the skill and intelligence of Prof. M., as it would that of the Surgeon, who should appeal to the feelings of his patient to enable him to distinguish between the artery he was ligating and its attendant nerve. And without any attempt to show the worthlessness of a guide, predicated on the feelings of a woman, whose "mind is strained to the highest tension," and who requires "good intervals of rest," for her "moral relief," I am inclined to believe with Prof. Simpson, that if this

"really formed the safest and most trustworthy guide in the operation," and was honestly "worth a thousand dogmas and precepts," Prof. M. would not have omitted all allusion to it in his published works; the more especially, as Simpson observes, with quiet sarcasm, that "all other authors omit notice of it." I cannot refrain, however, from calling attention to the case cited by Prof. Meigs on page 551 (*Obstetrics; the Science and Art*, 1852), and asking if such accident would have been possible in the motionless slumber of Anæsthesia? and if not, what becomes of the charge of undesirability? Here, the patient's ability to dictate to the Surgeon by her sensations, was, it would seem, a thousand times more than counter-balanced by the conscious endurance of such pangs as to cause her to "throw up the pelvis," so "violently and unexpectedly," that the operator, (Prof. Meigs, himself,) was unable to prevent the "very severe laceration of the perineum," from which effects, we are candidly told, it took her three weeks to recover. Would Prof. M. have us believe, that his knowledge of the anatomy of a portion of the human frame, he has made a life-long study, was so imperfect and unreliable, that without the "safest and most trustworthy diagnosis" of his patient's sensations, he would inflict such laceration and injury, in the introduction of the forceps, as it would require three weeks to recover from? If not, is this charge tenable? Indeed, are not both arguments, and the charges they are meant to sustain, disproved over and over again, by Prof. Meigs' own writings?

Not doubting the answer, I pass to the charge of drunkenness as a condition of chloroformal insensibility—a charge preferred by Ramsbotham in 1851, in the strongest manner, and urged with much eloquence and wealth of expletive; subsequently considerably modified, and pruned of its appeals to "moral obligations," etc., but adopted by Prof. Meigs in his edition of '52 in the following language: "But I cannot avoid the feeling of astonishment which seizes upon me when I read the details of cases of midwifery, that have been treated during the long, profound *drunkenness* of etherization."

It is worthy of remark, that Prof. Meigs makes but the most cursory allusion to this objection in his letter to Prof. Simpson in 1848, and the "feeling of astonishment" does not appear to have seized upon him till 1852, or subsequently to Prof. Ramsbotham's preferment thereof. Dr. Murphy, in his "Chloroform in Childbirth," says: "The Anæsthesia of Chloroform has not the least resemblance to drunkenness; they have not a symptom in common." Setting this authority aside for the time, however, and admitting for the sake of argument, that the charge is true, how much more applicable is it to results produced by chloroform, than to the same produced by opium, camphor, (which Prof. M., with Dr. Physick, agrees "to have been made for women") cannabis indica, hyoscyamus, or any other of the large and very useful classes of inebriants, soporifics and deliriants; and yet Prof. Meigs would be very careful how he placed any one of these articles in his *Index Expurgatorius.* Nay, with what a lavish disregard of Mr. Gough and individual consistency, does he prescribe "good Bordeaux wine" and a "small glass of sherry or port, at dinner," without a thought of the results inevitably accruing?

I do not know that I am able to point out the difference between the insensibility from whisky, gin, brandy, wine and beer, and that from ether and chloroform. Prof. Meigs says "no reasoning—no argumentation is strong enough to point out the ninth part of a hair's discrimination between them—*except* that the volatility of one of the agents, or its diffusibility as a stimulant narcotic, enables it sooner to produce its intoxicating effect, which is sooner recovered from in one case, than in any other of the use of an intoxicating drug." Add to this, that chloroform begets a positive disgust to its vapor, by use, and with this exception, I profess to be abundantly satisfied. For I claim that the insensibility of alcoholic intoxication is its least exceptionable feature; and if this be the sole point of resemblance between chloroformal and alcoholic intoxication,—if the use of the former agent, shall produce no appetite to be gratified at the expense of health, honor, happiness, friends, reason, even life itself, as does the use of the latter, and which is the true objection to the use of all alcoholic fluids—"good Bordeaux wine," "sherry and port" included,—if this be the only point of resemblance, then the argument is equally valid against all the agents of the Materia Medica, whose province it is to annul pain, obtund consciousness, and produce sleep. Dr. Murphy, quoted above, contends that the argument is as valid against Nature herself—since she deprives us of consciousness each night, and "when the agonies of childbirth become so intense as to be no longer tolerable, sometimes induces that complete state of both unconsciousness and anæsthesia, called puerperal convulsions."

Under the head of Impropriety, comes also, the religious objections, presented by Prof. Meigs in the following language: "I have by no means said what I am inclined to say, as to the doubtful nature of any processes that the physician sets up, to contravene the operation of those natural and physiological forces, that the Divinity has ordained us to enjoy or to suffer."

Without endorsing Prof. Simpson's elaborate and scholarly argument in answer, based, as it is, on philogical erudition which I do not possess, and which is, to say the least, made questionable, by the translation and commentary on the passages of Scripture discussed by Prof. Simpson, in the recent works of the eminent Dr. Kalisch, and by Prof. Noyes, of Cambridge, both high authorities in Hebrew, I do not hesitate to avail myself of reasonings and facts cited in support of other views.

Most prominently in this connection, arises the question, what is a truly Natural Labor; and if we accept the definition of Prof. Miller, "one, in a good degree, free from such pain as we are accustomed to witness," I am unable to see the contravention of any Divine ordinance in the induction of any process which shall annul that extra degree of pain which makes labor unnatural; and which is the joint result of the unnatural condition of the woman of to-day, produced by "the hot-beds of civilization," and the increased development of the skulls of civilized races; a development, not participated in by the pelves of the women, if indeed, they be not positively contracted by modes of dress, habits of life, etc. How else can we account for the immunity of the female barbarian from such pain as her civilized sister is bade to endure as a Divine decree?

To sum up the counts of the first head, and our answers, we have first: Labor-Pains alleged to be conservative, salutary and desirable; and an essential element of the function of parturition. Disproved by the brief analysis of the office of Pain, and by extracts from Prof. Meigs' own writings. *Per contra*, I have shown these pains to be useless, objectionable, and, in some cases, positively pernicious; and the distinction between Pains created by Prof. Meigs to meet the emergency, as fanciful and unphilosophical—doing more credit to his ingenuity, than to his scientific reputation.

II. The inability of the anæsthetized patient to counsel the operator in the use of the forceps. Very fully overbalanced by her restlessness, unanæsthetized, causing accidents painful and tedious of recovery—to say nothing of the ignorance and untrustworthiness, implied in the operator who should rely on such a guide, or of its unreliability, owing to the mental condition of the patient at such times, and the variety of degrees of endurance in various women.

III. Moral objections to Anæsthesia, on the ground of its similarity to alcoholic drunkenness. Similarity shown to be only in a minor physical point, and in nowise bearing a resemblance to the immoral sequelæ of alcoholic intoxication.

IV. Religious objections to the process, as contravening the primal curse. Shown to arise from a narrow, unphilosophical view of the subject—which ignores the facts that civilization has thus far, increased the size of the fœtal head, and reduced the bony system of the mother in a varying ratio; that barbarous and semi-civilized tribes, and now and then, individual cases in the most highly-civilized races, are exempt from that degree of suffering in labor, which makes the process, according to Prof. Miller, unnatural. Much is omitted in this, as in other sections, from want of time, and fear of prolixity, but I cannot refrain from alluding to the fact mentioned in Exodus I, 19, as pertinent here: "And the midwives said unto Pharoah, Because the Hebrew women are not as the Egyptian women; for they are lively, and are delivered ere the midwives come in unto them." A Scriptural proof of much more weight than that cited by Prof. Simpson of Adam's sleep while his rib was removed—and which latter, by the way, had occurred to me before seeing Prof. S.'s allusion to it; but was set aside, notwithstanding the subsequently met testimony of Luther,—that the sleep was induced to prevent Adam feeling any pain—on the ground that the incident occurred before the fall, and before Sin, Pain and Death had entered the world—a fact, which all commentators, so far as I know, overlook.

But to return from this digression. I take up the counts of the second head, viz: *The danger and evil results of Anæsthesia.*

It would be too tedious to enumerate all the evil results that have been attributed to this practice:—flooding, convulsions, tardy recoveries, effusions, poisoning of the child, even puerperal mania, and idiocy, with many other equally monstrous and absurd charges—the simple and conclusive refutation of which is to be found in the entire absence of any kindred charge in Surgical Anæsthesia, and a detailed disproof of which is here unnecessary, if not impracticable.

The final and gravest objection to Anæsthesia in Midwifery is, that of Danger of Fatal Results, one indeed, not strongly pressed at the present day, but still embodied in Prof. Meigs' latest editions; and to give a show of reason for which, the learned writer makes use of a piece of special pleading, which would not discredit the reputation of the far-famed Philadelphia bar, to-wit: "I readily hear, before your voice can reach me across the Atlantic, the triumphant reply that an hundred thousand have taken it without accident. I am a witness that it is attended with alarming accidents, however rarely. But should I exhibit the remedy for pain to a thousand patients in labor, merely to prevent the physiological pain, and for no other motive—and should I in consequence destroy only one of them, I should feel disposed to clothe me in sackcloth and cast ashes on my head for the remainder of my days. What sufficient motive have I to risk the life or death of one in a thousand, in a questionable attempt to abrogate one of the general health conditions of man?" This is copied from his letter to Prof. Simpson, before referred to. It will be observed that Prof. Meigs starts out with the assumption that it had been used in an hundred thousand cases without accident; Prof. S. in his letter, to which this is a reply, distinctly states that "no accidents have as yet happened under its use, though *several* hundred thousand must have already been under the influence of chloroform." Prof. Meigs then goes on to reduce the number of cases to one thousand, and finally winds up with the assumption that one death occurs in a thousand, and queries what right he has to risk that—a mode of argument befitting a jury lawyer more than a grave professor discussing a medical question. The objection and the argument, however, is best met with the stubborn fact that despite the jealous watchfulness of Meigs, Ramsbotham, Arnott, Lee, and the numerous lesser lights of its opponents, but two deaths from chloroform, not one from ether, and not one from either chloroform or ether, administered by a medical man, in midwifery, are yet recorded. And the use of these agents may be stated in round numbers at 20,000,000 cases in the United States, Great Britain, France and Germany, in the last twelve years!

Can any more convincing proof of the groundlessness of the charge of Danger, be produced or demanded?

Prof. Ramsbotham has, indeed, claimed to have discovered one fatal case by the use of chloroform in the hands of a physician; but the proof is so decidedly against this, as the cause of death, that no one else has been found willing to cite it; and I doubt much if Prof. R. himself, would now urge it. Dr. Snow, to whom I refer for a report of the case, arrives at the conclusion that chloroform had nothing, whatever, to do with the death; inasmuch as the patient had been successfully anæsthetized in two previous labors, had fully recovered from the chloroform, in the one before her death, and was comfortable, conversed with friends, etc., for an hour and a half before feeling any distressing symptoms, and finally that these (symptoms) "did not coincide at all with the known effects of chloroform."

Of the two other cases, the exceptions I have mentioned above to the innocuousness of Anæsthesia in Midwifery, both occurred in the hands of non-professional persons. The first, in 1855, in England, was the result of its induction by the patient herself, against the advice of her attendant; five drachms were used and the inhaler (a handkerchief), remained in close contact with the patient's face until death ensued. The other case occurred in Scotland, in 1858; the chloroform was administered by her husband; "on giving it probably for the fourth time, she threw herself violently back, gave a gasp or two, a slight gurgle was heard in her throat and respiration and the pulse instantly ceased."

I have quoted details in these cases (the former from Dr. Snow's report, the latter from Dr. Campbell's statement to Dr. Lee), for the purpose of future reference; meanwhile in view of the fact that not a single death can be traced to the use of chloroform or ether administered by medical men in (estimated) 20,000,000 obstetrical cases I assert that this last, most serious charge is utterly devoid of foundation; and defy a similarly favorable record to be produced for any other equally powerful agent of the Materia Medica.

To resume then; if anything, I have proved,

1st. That the hostility to Anæsthesia in midwifery is due, mainly, to the efforts of a learned, eloquent and influential opponent, early committed to his antagonistic position, and too fixed in opinion to even re-examine the subject.

2d. That the objections are alike unsound in theory, and unfounded in fact; in proving which latter I have also shown incidentally that

3d. Anæsthesia in midwifery is desirable, useful and proper; for that it annuls pain, which, being an evil, abnormal, in this instance useless, and productive, in itself, of mischievous consequences, even to death itself, is the legitimate object of the physician's hostility. And,

4th. That it is perfectly safe, and free from annoying or dangerous sequelæ.

In the foregoing pages I fear I may have, now and then, appeared to overstep the bounds of that deference and respect, which none more readily than myself would observe, as the just meed of the tutor from his pupil, of Learning and Wisdom from Ignorance and Inexperience. And yet no writer whom it has been my good fortune to peruse, more uniformly or more strenuously insists on the right and duty of the student to criticize and dissect the precepts of his author, than Prof. Meigs ; and in no other section, than in this upon *Etherization*, does he speak more directly to the student, or leave more questions for the student to examine and settle for himself. In availing myself of this right and duty, I have been actuated solely, I trust, by a desire for the truth, influenced only by a wish to render some service, however slight, to the cause of humanity, in the abolition of human suffering ; and I desire, before closing, to place in the same record, an expression of my deep sense of respect and admiration of Prof. Meigs, as a devoted, enthusiastic lover of his profession, a sedulous, untiring student, and a writer, the most fascinating and instructive.

In closing this effort, I cannot overcome a feeling of disappointment and regret at the crude, disjointed character of an appeal in behalf of a practice, which, though its array of facts is overwhelming and indisputable, still needs all the eloquence and genius which its opponents have lavished in its assault, to resist their attacks, and counterbalance the sentiment they have created against it.

The history of Anæsthesia presents an anomaly, most singular and deplorable, in its comparative exclusion from midwifery, while so universally resorted to in surgery. And the fact that, of all the physicians whom I have questioned upon the subject, I have not met one who refuses to employ these agents when insisted upon by patients in labor, is *prima facie* evidence, to me, that the mind of the medical profession is not clear and decided upon the subject. And yet see the dilemma this places them in!

If Prof. Meigs and his followers be right in their views, the exhibition of chloroform and ether is pernicious and improper to a degree which warrants its positive denial; and the physician who, holding such views, yields to the solicitation of his patient, is as morally guilty of compounding a felony, even though no bad results ensue, as is the professional outcast, who consents to produce an abortion, under the same influence.

If he withholds it generally, from a confused, uncertain idea that it is dangerous, and yet yields to the pleadings of his patient, upon whom he fancies he thus throws the responsibility, then I claim that he is derelict in his duty, and in the fullest, truest sense, is not a Physician, but a tamperer with the holy science. And I envy not that man, be his professional position what it may, who through indecision or lack of investigation, is content to stand idly by the bedside, a dispassionate witness of pangs indescribable, agony incomparable, at a time when the highest, holiest, most mysterious sympathies of our nature should be evoked—the advent of an immortal soul into the world.

If the miraculous vapor be Divine in its mission at any one time more than another, it is not when the crushed and mangled victim lies helpless upon the operating table, when the cruel steel is poised in hand, previous to its terrible passage through the fair, white skin, the shrinking muscle and the quivering nerve. But when, with sorrow greatly multiplied, they that travail in child-birth, through this, the triumph of man's skill and study, are relieved of the curse, then, indeed, is man more godlike, and Anæsthesia a true *Donum Dei!*

### NOTE.

I deem it proper, in submitting the above, to state that I had contemplated, originally, a very different treatment of the subject, viz: An examination of the Physiology of Anæsthesia, with a view of elucidating the phenomena of its occasional fatal results. And to this end I had devoted considerable time and research to the Necrology of Anæsthesia; when, finding the subject beyond my depth, and demanding an amount of labor in study and experimentation, which the duties of the lecture term forbade, I determined upon the course, the results of which are herewith given, with no little diffidence and misgiving.

My first plan involved the collection and analysis of all the fatal cases on record—something under one hundred—but scattered *passim*, as they are over the current literature of twelve years, the undertaking was greater than it at first seemed; and was rendered doubly difficult by the very incomplete condition of the Medical Libraries of this city. Another source of information—that by correspondence—was only partially availed of, when I saw the reasons for changing my design.

I cannot, however, forego this opportunity (limited, though its publicity may be, by the dusty obscurity of the shelves of the College Library,) of acknowledging the kindness of those gentlemen who have favored me with the results of their own experience; chief among whom, allow me to gratefully mention the worthy colleague of Prof. MEIGS,—SAMUEL D. GROSS—two names which have received the highest enconiums of the profession, as first among the foremost in Obstetrics and Surgery.

I take the liberty of appending a copy of Prof. Gross' letter:

PHILADELPHIA, *Dec.* 15*th*, 1860.

DEAR SIR:—

I answer your letter of the 10th instant, with much pleasure.

You ask me, in the first place, why I "do not inhibit the exhibition of Chloroform in cases of organic lesions of the heart and lungs, more especially, the former." To this I reply: first, because I have never witnessed any bad effects from the use of it under such circumstances; and secondly, because there can be no impropriety in giving such a remedy when the malady is not so great as, in itself, to forbid operative interference. Chloroform, if of good quality, and judiciously administered, never produces any serious influence upon the action of the heart and lungs. The rule is, always to promote the free introduction of air into the respiratory passages.

I answer, in the second place, that no fatal case from the effects of chloroform has ever come under my observation. In one instance—that of a lad mentioned in my *Surgery*—the patient was nearly asphyxiated from an over dose of chloroform from inhalation while in the recumbent posture; and in another, he was nearly an hour in recovering from the effects of the remedy, although he was kept constantly on his back, both during and after the operation. Doubtless, the patient should never be permitted to sit up during an operation when he takes chloroform; at all events, my invariable practice is to make him lie down until the effects of the anæsthesia have, in great degree, if not wholly, disappeared.

There are, according to my experience, five cardinal rules for the administration of chloroform; and when they are properly observed, there is, I presume, little, if any, danger to be apprehended from the use of the remedy. These rules are as follows:

1. The selection of an unexceptionable article, such, for example, as that manufactured by Dr. Squibb. Formerly I used nearly altogether the Edinburgh chloroform, but I believe that the American is quite as pure, and consequently, as safe.

2. The free admission of atmospheric air along with the anæsthetic; the administrator giving the subject his undivided attention while the inhalation is in progress.

3. An empty state of the stomach, and a moderately empty state of the bowels. For this purpose, the patient should not take any food for six or eight hours, prior to the operation. If the bowels are loaded, a purgative should be administered the previous evening.

4. An absence of all constriction of the chest and abdomen, so as to admit of the free play of the diaphragm and intercostal muscles.

5. Complete recumbency during the administration, not allowing the patient to rise, on any account, whatever.

If these rules be carefully observed, my conviction is, that it would be extremely difficult to kill a person with chloroform, even when there is considerable organic lesion of the heart and lungs.

With kindest regard,

I am, dear sir,

Very truly, your friend,

Dr. F. W. Reilly, *Chicago.* S. D. Gross.

To the above rules nothing can be added, it seems to me, save a more emphatic repetition of No. 2. I feel quite convinced, in my own mind, and design, at some future time, to attempt the proof, by data already partially collected, that deaths by the inhalation of chloroform, may be divided into two, or at most, three classes; the first of which, and the most important, includes those resulting from a spasmodic closure of the glottis, produced by the action of the undilute vapor—a condition not difficult to realize in the recumbent position, with the pure vapor falling in a heavy volume through the half-inch or so of space most administrators seem to think sufficient; and I am led to suggest, after repeated experiment, the doubt whether, after all, the position on the back is the safest under all circumstances; and to query if it will not, eventually, be found advisable to place the patient upon his *side*, in the first steps of the process. Chloroform vapor is very much heavier than atmospheric air; and though diffusible, is not permeable—by which, I mean, that though it will mix with atmospheric air, in all proportions, yet this latter will not penetrate a stratum of the vapor, without mechanical disturbance. Any one may test for himself, without trouble, the effect of the first inhalation, and the marked comparative ease with which it subsequently proceeds, until it becomes necessary to renew the supply. The irritation subsides with each succeeding inspiration, partially owing to the force of habit, but mainly because the vapor is more and more dilute with every additional breath, until entirely exhausted. While the supply is being renewed, a few seconds intervene, during which no vapor is inhaled at all,—the glottis returns to its normal sensitive condition, and is as liable to spasmodic closure against the offending agent as at first. And, now it seems to me, is the true moment of danger: The administrator, emboldened by this first experience, stimulated, too frequently, by his own desire to cut short the noisy, intoxicated stage, and by the impatience, often, of the Surgeon to proceed with the operation, applies the freshly saturated cloth, sponge, or what not, still more closely to the patient's nostrils. Regarding the struggles of the latter, should he be able to make any, as merely the manifestation of his intoxication or perverseness, he follows every motion of the head closely, or holds it fixed with his hand; the heavy column of chloroform falls pure and unmixed into the nostrils; respiration ceases for a few fatal moments, and when the spasm relaxes, the nerve centres are overwhelmed, the circulation, already languid and charged with carbonic acid, bereft of its stimulus, flags—and the patient is dead! These, I have no doubt, were the phenomena in the case related by Dr. Campbell, referred to in the *Thesis;* also in the first Bartholomew Hospital case, and in many others whose details I have carefully studied. I am further convinced of the soundness of this view from personal experiment,—of which more, possibly, anon.

The practical deductions from this are, that "*free admission of atmospheric air along with the Anæsthetic,*" *is of the* FIRST AND UTMOST IMPORTANCE. That the undivided attention and intelligence of the administrator is necessary; no consideration of expense either of time or for chloroform should be allowed to enter into the calculation; better far, spend fifteen minutes more with each of a thousand patients, if you thereby insure their individual safety, than save that time to nine hundred and ninety-nine, at the expense of the life of the thousandth.

To insure this *gradual induction*, I believe the position on the side, most favorable; the nose being, say, two to two and a half inches from the pillow, the inhaler held above the line of the nostrils, at a distance, never less than an inch and a half, and better two inches above. The vapor is thus not alone mixed with the atmosphere by the mechanical disturbance of the breath, driven through it, but only that vapor obtains access which is *drawn in*, none being allowed to *fall* into the mouth and nostrils of its own specific gravity, as is the case when the position on the back is assumed. With these precautions, the induction of Anæsthesia may require double the present ordinary time; but its safety must be materially increased.

